# Natural Compounds in Cancer Therapy: Revealing the Role of Flavonoids in Renal Cell Carcinoma Treatment

**DOI:** 10.3390/biom15050620

**Published:** 2025-04-25

**Authors:** Zixuan Chen, Min Liu

**Affiliations:** Department of Urology, Tongren Hospital Shanghai Jiao Tong University School of Medicine, Shanghai 200336, China; czixuan2023@sjtu.edu.cn

**Keywords:** renal cell carcinoma, natural products, flavonoids, drug discovery, combination therapy

## Abstract

Renal cell carcinoma (RCC) is the most lethal malignancy of the urinary system, with limited treatment options due to drug resistance and the adverse effects associated with current therapies. This review aims to systematically examine the therapeutic potential of flavonoids, which are natural polyphenolic compounds possessing anti-inflammatory, antioxidant, and anticancer properties, in the context of RCC treatment. We summarize the anticancer activities of 26 natural flavonoids, classified into six subclasses, and explore their mechanisms of action, including the inhibition of tumor cell proliferation, migration, and invasion, as well as the induction of apoptosis, autophagy, and ferroptosis. Particular attention is paid to their modulation of key signaling pathways such as the JAK/STAT3, PI3K/Akt/mTOR, and miRNA-related axes, including miR-21/YAP1 and miR-324-3p/GPX4, providing a molecular basis for their anti-RCC activity. We also address several pharmacological challenges that limit the clinical application of flavonoids, including poor bioavailability, metabolic instability, and potential toxicity. Emerging solutions such as novel flavonoid derivatives, advanced drug delivery systems, and rational combination therapy strategies are also discussed. Current clinical evidence, including a phase II trial of flavopiridol in advanced RCC, highlights the potential but also the need for further validation. In conclusion, flavonoids offer a promising approach to improving RCC treatment. Future research should focus on optimizing their therapeutic efficacy and ensuring their safe clinical translation, with the goal of achieving personalized and minimally invasive cancer therapies.

## 1. Introduction

Renal cell carcinoma (RCC) is the most common primary malignant tumor of the kidney, accounting for approximately 2–3% of all malignancies worldwide. RCC is also one of the most prevalent and lethal genitourinary cancers, with a high incidence and mortality rate. According to recent GLOBOCAN data, in 2022, there were over 430,000 new cases of RCC and more than 150,000 associated deaths worldwide [1]. For early-stage localized RCC, surgical resection remains the standard treatment, offering favorable therapeutic outcomes. However, nearly one-third of patients present with distant metastasis at diagnosis, precluding the possibility of curative surgical intervention. The emergence of immunotherapies, such as immune checkpoint inhibitors (ICIs), and targeted therapies, such as tyrosine kinase inhibitors (TKIs), has revolutionized the treatment of advanced and metastatic RCC. Numerous clinical trials have demonstrated that targeted therapies and immunotherapies significantly improve overall survival (OS) and progression-free survival (PFS) in metastatic RCC patients. However, challenges remain, particularly with the development of drug resistance and the occurrence of non-responses, which continue to complicate RCC management [2]. For instance, sunitinib, a first-line TKI for RCC, commonly induces drug resistance within approximately six months of treatment. Similarly, nivolumab (an anti-PD-1 antibody) combined with ipilimumab (an anti-CTLA-4 antibody) represents a first-line therapy for RCC, benefiting nearly half of all patients, though approximately 20% exhibit primary resistance to this regimen [3]. Furthermore, both targeted therapies and immune inhibitors are frequently associated with adverse effects, such as gastrointestinal disturbances, hepatic toxicity, and bone marrow suppression, which can limit long-term adherence [4]. These challenges—such as the inapplicability of surgical treatment for patients with advanced and progressive RCC, and the propensity of targeted and immunotherapies to induce resistance, non-responsiveness, and adverse events—underscore the urgent need for novel therapeutic agents that can either provide safer and more effective treatment options or enhance the efficacy of existing targeted and immunotherapies in RCC.

Natural products, comprising bioactive compounds derived from plants, animals, microorganisms, and other natural sources, include classes such as alkaloids, polysaccharides, flavonoids, lignans, polyketides, saponins, tannins, and terpenes [5]. The therapeutic value of these compounds has been widely recognized and extensively studied, particularly since the advent of modern medicine. A notable milestone in this field was the extraction of morphine from poppy plants in 1804, marking a pivotal advancement in the use of natural products as therapeutic agents [6]. Over time, natural-product-derived drugs—such as ephedrine for asthma, artemisinin for malaria, and penicillin for bacterial infections—have significantly influenced the evolution of medical treatments [7]. In oncology, the potential of natural products has been especially pronounced. Active compounds sourced from nature have demonstrated remarkable anticancer activity, with well-defined chemical structures and relatively high safety profiles, making them promising candidates for cancer therapy [8]. By 2019, nearly half of the 247 new anticancer drugs approved by the U.S. Food and Drug Administration (FDA) were derived from natural products [9].

Among these bioactive substances, flavonoids are particularly abundant in the plant kingdom and are widely distributed in flowers, leaves, and other plant parts [10]. Structurally, they consist of a fifteen-carbon skeleton arranged in a C6–C3–C6 configuration, comprising two aromatic rings (A and B) linked by a three-carbon bridge that often forms a heterocyclic ring (C-ring) [11]. Based on variations in the C-ring and the degree of hydroxylation, methylation, glycosylation, and other substitutions, flavonoids are classified into several subclasses, such as flavonols, flavones, flavanones, flavanols, anthocyanins, isoflavones, and chalcones [12]. The biological activity of flavonoids is largely influenced by their structure. Key structural features that impact their activity include the number and position of the hydroxyl groups, the degree of conjugation within the molecule, and the presence of glycosidic linkages. These attributes determine their ability to interact with cellular targets, influence signaling pathways, and exert antioxidant, anti-inflammatory, and anticancer effects. For instance, flavonoids containing di-OH 3′, 4′, a double bond at C2–C3, and a carbonyl at the C4 position have been shown to exhibit significant anticancer properties [13]. Extensive research has demonstrated the significant role of flavonoids in cancer treatment [14]. For instance, chrysin, a flavonoid derived from *Passiflora* L., has exhibited anticancer activity in hepatocellular carcinoma, lung cancer, and melanoma [15]. Recent studies suggest that flavonoids also hold promise for treating RCC, as they may inhibit tumor growth, induce apoptosis, and enhance the efficacy of conventional therapies.

Although previous studies have explored the anticancer activities of natural products, the unique advantages of flavonoids have not been fully elucidated or summarized in the context of RCC, a malignancy characterized by high heterogeneity and therapeutic resistance. This review is the first to systematically summarizes the mechanisms of action of flavonoids against RCC. Additionally, it focuses on discussing strategies to enhance the bioavailability of flavonoids and address clinical challenges such as treatment resistance in RCC, with a focus on improving their clinical translation. This comprehensive analysis not only updates the molecular pharmacological understanding of natural products against RCC but also provides innovative perspectives to overcome the current bottlenecks in targeted and immune therapies.

## 2. Literature Search Strategy

A comprehensive literature search was conducted to identify relevant studies published up until November 2024 that investigated the effects of flavonoids and natural products in the treatment of RCC. The databases searched included PubMed, Web of Science, Scopus, and CNKI. The following keywords and their combinations were used: “natural products”, “flavonoids”, “renal cell carcinoma”, “cancer”, “derivatives”, “resistance”, “nanomaterials”, and “clinical trials”. The Boolean operators “AND” and “OR” were applied to refine the search strategy.

The articles were assessed for eligibility based on predefined inclusion and exclusion criteria. The inclusion criteria were as follows: (1) studies focusing on the pharmacological effects of flavonoids or their derivatives on RCC; (2) studies published in English or Chinese; and (3) articles reporting in vitro, in vivo, or clinical evidence. The exclusion criteria included (1) studies focusing on other cancer types without specific relevance to RCC; and (2) studies investigating compounds that were not classified as flavonoids.

Ultimately, 112 articles met the inclusion criteria and were included in this review. Data from these studies were synthesized narratively to provide a comprehensive understanding of the therapeutic potential, molecular mechanisms, and clinical perspectives of flavonoids in RCC treatment.

## 3. Mechanisms of Anticancer Action of Flavonoids Against RCC

Numerous flavonoids have demonstrated potent therapeutic effects against RCC. This section provides an overview of the mechanisms underlying the anticancer properties of specific flavonoids in the context of RCC (Table 1).

### 3.1. Flavones

Flavones are a major subclass of flavonoids that are widely distributed in fruits, vegetables, and medicinal herbs. They exhibit a broad range of pharmacological properties, including antioxidant, anti-inflammatory, and anticancer activities. This subsection summarizes the antitumor mechanisms and therapeutic potential of representative flavones in RCC treatment.

Luteolin, a flavonoid originally isolated from *Reseda lutea* L. and present in various plants like *Artemisia vulgaris* L., honeysuckle, and chili peppers, is known for its anti-inflammatory and anticancer properties [53]. Luteolin has been shown to exert its anticancer effects in RCC by inducing apoptosis and ferroptosis. A critical mechanism of action involves the modulation of the tumor necrosis factor-related apoptosis-inducing ligand (TRAIL) pathway, which selectively induces apoptosis in cancer cells [16]. Despite TRAIL’s limited efficacy due to resistance mechanisms such as the absence of TRAIL receptors, FLIP overexpression, and activation of the Akt and STAT3 pathways, luteolin has been shown to overcome these resistances [54]. In vitro studies have demonstrated that luteolin targets p-Akt and p-STAT3, downregulates FLIP, and sensitizes RCC cells to TRAIL-induced apoptosis [17]. Furthermore, luteolin promotes ferroptosis by upregulating heme oxygenase-1 (HO-1), resulting in heme degradation, increased free iron ions, mitochondrial dysfunction, and elevated reactive oxygen species (ROS) levels, ultimately leading to ferroptosis in RCC cells.

Scutellarin, a flavonoid compound derived from *Scutellaria* L. and *Erigeron breviscapus*, exhibits various medicinal properties, including neuroprotective, anti-inflammatory, and antitumor effects [55]. Scutellarin has been shown to inhibit RCC cell invasion by reducing the expression of MMP-2 and MMP-9, which are essential for the metastatic spread of cancer cells [18]. Furthermore, scutellarin induces G0/G1-phase cell cycle arrest and promotes apoptosis in RCC cells. The underlying mechanism involves the upregulation of the tumor suppressor protein PTEN, which inhibits the PI3K/AKT/mTOR signaling pathway, a key regulator of cell survival and proliferation.

Apigenin, a natural flavonoid commonly found in vegetables and fruits such as celery and *Matricaria chamomilla* L., has been extensively studied for its anticancer effects across various cancer types, including liver cancer, gastric cancer, glioma and RCC [56,57]. Apigenin has been shown to inhibit RCC cell proliferation by inducing DNA damage and regulating cell cycle progression [19]. Studies indicate that apigenin causes G2/M-phase cell cycle arrest by downregulating the expression of cyclins (A, B1, D1, D3, E) and CDK1 [20].

Nobiletin, a flavonoid abundant in citrus fruits such as oranges and lemons, exerts its anticancer effects by reversing hypoxia-induced epithelial–mesenchymal transition (EMT) in RCC cells [21]. Under hypoxic conditions, RCC cells activate the NF-κB and Wnt/β-catenin signaling pathways, promoting cell migration and invasion. Nobiletin blocks this process and suppresses RCC progression. Further studies have revealed that nobiletin targets multiple signaling pathways, including SRC/AKT, JAK2/STAT3, and PI3K/Akt, to inhibit RCC cell proliferation, migration, and invasion [22,23]. Additionally, nobiletin enhances RCC sensitivity to the CDK4/6 inhibitor palbociclib by targeting SKP2, suggesting its potential for combination therapies in RCC [24].

Diosmetin, a flavonoid extracted from plants such as acacia and lemon, has been extensively studied for its biological activities, including anti-inflammatory, antioxidant, and anticancer effects [25]. Qiu et al. [58] revealed that diosmetin inhibits cell viability and induces apoptosis in RCC cells, with minimal effects on normal renal tubular epithelial HK-2 cells. Furthermore, diosmetin disrupts the PI3K/Akt pathway by inducing p53 activation, which in turn leads to the downregulation of AKT phosphorylation.

Hispidulin, a flavonoid isolated from plants like snow lotus and *Grindelia argentina* [59], has demonstrated various biological activities, including anti-inflammatory, neuroprotective, antioxidant, and anticancer properties [60]. In RCC, hispidulin enhances TRAIL-induced apoptosis in RCC cells by stabilizing the pro-apoptotic protein Bim through the CaMKKβ/AMPK/USP51 signaling pathway. This action sensitizes RCC cells to TRAIL-mediated cell death, positioning hispidulin as a potential therapeutic agent in RCC therapy [26].

Morusin is a natural compound extracted from *Morus alba* L., and its medicinal value has been the subject of ongoing exploration. Currently, morusin is recognized for its anti-aging, anti-inflammatory, and antitumor effects [61]. Yang et al. [27] showed that morusin inhibits RCC cell proliferation and migration via the MAPK signaling pathway. Furthermore, morusin induces G1-phase cell cycle arrest and promotes apoptosis, contributing to its anticancer effects in RCC.

Jaceosidin, a flavonoid isolated from *Artemisia vestita* Wall. ex Bess., is widely known for its anti-inflammatory and antioxidant properties [62]. Additionally, jaceosidin plays a significant role in the treatment of various cancers, including RCC [63]. Woo et al. [28] found that jaceosidin disrupts mitochondrial integrity in RCC cells, leading to a loss of mitochondrial membrane potential, activation of the pro-apoptotic protein BAX, and downregulation of the anti-apoptotic protein Mcl-1.

Eupafolin is a flavonoid natural product primarily found in *Rudbeckia hirta* L. Extensive research has demonstrated its potential anticancer properties [64]. Han et al. [29] found that neither eupafolin nor TRAIL alone induced significant apoptosis, but combination therapy markedly enhanced apoptosis in RCC cells. Eupafolin was shown to downregulate Mcl-1 expression at the post-translational level in a cathepsin-S-dependent manner. Overexpression of Mcl-1 blocked apoptosis induced by the combination treatment. Additionally, eupafolin upregulated Bim expression via activation of AMPK, which inhibited proteasomal degradation and stabilized Bim. Knockdown of Bim expression using siRNA prevented the apoptosis induced by eupafolin and TRAIL. Moreover, combined treatment with eupafolin and TRAIL significantly reduced tumor growth in xenograft models, suggesting its potential therapeutic application.

Eupatilin is a flavone compound primarily isolated from *Artemisia argyi* Lévl. et Vant. It plays an important role in the treatment of diseases such as gastritis, periodontitis, and RCC [65]. Zhong et al. [30] demonstrated that eupatilin inhibits the proliferation and migration, and induces apoptosis, in RCC. It was found that eupatilin downregulates miR-21, which is often overexpressed in RCC and linked to tumor progression. miR-21 was shown to directly target Yes-associated protein 1 (YAP1), a key regulator of cell growth and survival. By reducing the miR-21 levels, eupatilin activates YAP1, which enhances apoptosis and suppresses cell migration. This study highlights the potential of eupatilin as a therapeutic agent in RCC treatment, particularly through the modulation of miRNA and signaling pathways such as the miR-21/YAP1 axis.

In summary, flavones exhibit multifaceted anticancer activities in RCC. Notably, although numerous flavones have been investigated for their therapeutic potential in RCC, many others remain unexplored. For instance, baicalin and baicalein, which are abundantly present in *Scutellaria baicalensis Georgi* alongside scutellarin, have demonstrated significant anticancer activities in various malignancies [66]. However, their mechanisms of action in RCC have yet to be elucidated. These knowledge gaps suggest that the full therapeutic value of flavones in RCC treatment remains far from fully realized, and future studies are warranted to uncover additional flavone candidates with promising efficacy against RCC.

### 3.2. Flavanones

Flavanones, a subclass of flavonoids predominantly found in citrus fruits and medicinal herbs, are recognized for their potent antioxidant, anti-inflammatory, and anticancer properties. In RCC, several flavanones have demonstrated therapeutic effects through modulation of key signaling pathways and oxidative stress responses.

Alpinetin is a flavonoid isolated from the ginger family plant *Alpinia katsumadai* Hayata, known for its various biological properties, including antitumor, anti-inflammatory, and antiviral effects [67]. In RCC, Guo et al. [31] employed network pharmacology and experimental analysis to demonstrate that alpinetin inhibits RCC cell proliferation and migration while promoting cell apoptosis. Regarding its mechanism of action, they found that the PI3K/AKT/mTOR pathway plays a crucial role in alpinetin’s anti-RCC effects. Additionally, in a mouse model, alpinetin was shown to reduce the tumor volume and weight and to inhibit the expression levels of the p-PI3K, p-Akt, and p-mTOR proteins within the tumors.

Hesperidin, a flavonoid primarily isolated from citrus fruits, has demonstrated a range of biological properties, including anti-inflammatory, antioxidant, and antitumor effects [68]. Siddiqi et al. [32] revealed that hesperidin significantly inhibits RCC progression by targeting the COX-2/PGE2 pathway. By reducing lipid peroxidation and increasing antioxidant levels, hesperidin effectively decreases oxidative stress in the kidney. Additionally, it downregulates COX-2, PGE2, and vascular endothelial growth factor (VEGF) expression. These findings suggest that hesperidin could be a promising therapeutic agent for RCC prevention.

2′-Hydroxyflavanone is a flavanone extracted from citrus fruits, such as oranges, which has demonstrated significant antitumor activity in various cancers [69]. Dalasanur et al. [33] demonstrated that 2′-hydroxyflavanone inhibits the growth of RCC through multiple mechanisms. It induces G2/M cell cycle arrest by downregulating cyclin B1 and CDK4, which suppresses cell division. Additionally, 2′-hydroxyflavanone reduces activation of the EGFR/PI3K/Akt signaling pathways, which are typically overactive in VHL-mutant RCC, thereby limiting cancer cell survival and growth. Furthermore, 2′-hydroxyflavanone acts as a glutathione S-transferase pi (GSTp) inhibitor, promoting oxidative stress within cancer cells while sparing normal cells. It also significantly lowers the VEGF levels, reducing tumor-driven angiogenesis and depriving the tumor of its blood supply.

Prenylnaringenin, an isoprenylated flavonoid isolated from *Humulus lupulus* L., exists primarily in two subtypes: 6-prenylnaringenin (6-PN) and 8-prenylnaringenin (8-PN). This compound exhibits diverse pharmacological activities, showing therapeutic potential in treating diseases such as pancreatic dysfunction, Alzheimer’s disease, and leukemia [70]. A study by Busch et al. [34] investigated the effects of 6-PN and 8-PN on renal cancer cells, demonstrating that both compounds inhibit cell proliferation in a concentration- and time-dependent manner.

In conclusion, flavanones exert their anti-RCC effects by targeting multiple oncogenic pathways, such as PI3K/AKT/mTOR, COX-2/PGE2, and EGFR, while promoting oxidative stress and inhibiting angiogenesis. Notably, hesperidin has also been reported to exert pharmacological effects by modulating gut microbiota metabolism [71]. In recent years, gut microbiota regulation has emerged as a promising strategy for RCC treatment [72]. Therefore, further exploration of the potential roles of hesperidin and other flavanones in modulating the gut microbiome to exert anti-RCC effects is warranted.

### 3.3. Flavonols

Flavonols are widely distributed in various dietary plants and are known for their diverse bioactivities, particularly in cancer prevention and therapy. In RCC, flavonols contribute to tumor suppression through the regulation of apoptosis, cell cycle progression, oxidative stress, and ferroptosis.

Kaempferol is primarily derived from the rhizomes of *Kaempferia galanga* L. but is also widely distributed in green plants such as tea leaves, broccoli, and hazelnuts [73]. The antitumor effects of kaempferol have been well documented in various cancers, including endometrial cancer [74], breast cancer [75], and colorectal cancer [76]. In RCC, kaempferol not only induces cell cycle arrest and apoptosis in RCC cells by targeting the EGFR/p38 pathway [35] but also inhibits RCC cell invasion and migration by targeting the AKT and FAK pathways [36], thereby effectively controlling the malignant progression of RCC.

Galangin, a flavonol abundantly found in the roots of *Alpinia officinarum* Hance, has demonstrated therapeutic potential in various diseases, including osteoarthritis, neurodegenerative disorders, and hypertension [77]. Its role in treating RCC has also been investigated. Han et al. [37] first demonstrated that galangin induces TRAIL-mediated apoptosis in RCC cells by inhibiting the expression of anti-apoptotic proteins such as Bcl-2 and Mcl-1. Subsequently, Cao et al. [38] further elucidated galangin’s anti-RCC mechanism, showing that it suppresses the expression of the antioxidant enzyme superoxide dismutase (SOD) while upregulating malondialdehyde (MDA) expression, leading to increased intracellular ROS levels.

Icariside II, also known as baohuoside I, is a natural flavonol compound abundantly found in *Epimedium koreanum* Nakai. In addition to its antidiabetic, neuroprotective, and anti-inflammatory effects on airway conditions [78], icariside II demonstrates notable antitumor properties [79]. Yu et al. [39] reported that icariside II inhibits RCC cell viability, proliferation, and migration, and it induces ferroptosis by upregulating miR-324-3p and downregulating GPX4. Moreover, using a xenograft mouse model, they confirmed that icariside II effectively suppresses RCC growth in vivo.

Icaritin, a natural compound extracted from *Epimediumbrevicornu* Maxim., has demonstrated substantial potential in cancer treatment [80]. Li et al. [40] confirmed that icaritin inhibits RCC cell proliferation and induces apoptosis by targeting the JAK/STAT3 pathway. Furthermore, in an RCC mouse model, they demonstrated that icaritin not only suppresses tumor growth but also inhibits angiogenesis by downregulating VEGF expression.

Gossypin, a flavonoid compound derived from *Hibiscus vitifolius* L., exhibits significant anti-inflammatory effects [81]. Recently, its pharmacological mechanism in treating RCC has been shown to involve the inhibition of inflammatory responses. Li et al. [41] demonstrated that gossypin suppresses tumor growth in a dose-dependent manner in a RCC xenograft mouse model. Specifically, gossypin reduced the phosphorylation of key proteins in the PI3K/Akt/mTOR signaling pathway, including p-PI3K, p-Akt, and p-mTOR. Moreover, gossypin treatment also targeted important inflammatory signaling molecules, such as NF-κB and STAT3, which are critical drivers of inflammation and tumor progression in RCC.

Overall, flavonols display significant antitumor efficacy in RCC by modulating key cellular processes and signaling pathways, including EGFR/p38, PI3K/Akt, JAK/STAT3, and ferroptosis-related targets. Their effectiveness in both in vitro and in vivo models highlights their potential for translational research and clinical application.

### 3.4. Isoflavones

Isoflavones, structurally similar to estrogens, are primarily found in legumes and exhibit strong antioxidant, anti-inflammatory, and anticancer effects. Their relevance in RCC treatment stems from their capacity to influence gene expression, epigenetic modifications, and miRNA-regulated pathways.

Corylin, an isoflavone extracted from *Psoralea corylifolia* Linn., has garnered attention for its anti-inflammatory and anticancer properties [82]. Yang et al. [42] discovered that corylin effectively inhibits cell proliferation by blocking the cell cycle at the G1 phase. Additionally, it induces apoptosis and suppresses cell migration and invasion. Moreover, corylin treatment suppresses RCC cells’ energy metabolism by impacting processes such as glycolysis and mitochondrial respiration. The effect is primarily mediated by downregulating the expression of the receptor for advanced glycation end products (RAGEs), which plays a critical role in the malignant progression of cancer.

Calycosin is a flavonoid compound that can be isolated from the dried root of *Astragalus membranaceus* (Fisch.) Bunge, with strong antioxidant, anti-inflammatory, and antitumor effects [83]. Zhang et al. [43] demonstrated that calycosin inhibits RCC proliferation and metastasis through the MAZ/HAS2 signaling pathway. It promotes apoptosis and reduces cell adhesion by targeting HAS2 expression, which is responsible for hyaluronic acid (HA) synthesis. Downregulation of HAS2 impairs RCC cell adhesion, limiting their ability to migrate and invade. Additionally, calycosin facilitates the ubiquitination and degradation of the transcription factor MAZ, further disrupting RCC progression. By reducing the MAZ levels, calycosin interferes with the extracellular matrix (ECM) integrity, which plays a critical role in cell migration and metastasis. In vivo studies have shown that calycosin significantly inhibits tumor growth without causing significant toxicity, suggesting it to be a promising therapeutic candidate for RCC.

Alpinumisoflavone is an isoflavonoid compound isolated from several plants, including *Maclura tricuspidata* Carrière, *Derris* Lour., and *Erythrina lysistemon* Hutch. It has shown therapeutic potential in treating various conditions, such as fibrosis, endometriosis, and cancer, including RCC [84]. Wang et al. [44] reported that alpinumisoflavone inhibits RCC growth and metastasis through modulation of the miR-101/RLIP76 signaling pathway and suppression of Akt activity. Alpinumisoflavone upregulates miR-101, which in turn downregulates RLIP76, resulting in reduced RCC cell proliferation and enhanced apoptosis. This mechanism highlights the potential of alpinumisoflavone as a therapeutic agent for targeting RCC by disrupting crucial pathways involved in tumor progression and metastasis.

Genistein is a natural isoflavone initially extracted from *Genista tinctoria* L., which is widely distributed in leguminous plants [85]. It has garnered significant attention due to its diverse pharmacological activities, including anti-aging, antioxidant, and anticancer effects [86]. In the treatment of RCC, genistein inhibits tumor progression through multiple mechanisms. First, long non-coding RNA HOTAIR binds to PRC2, typically suppressing the expression of the tumor suppressor gene ZO-1. Genistein counteracts this by inhibiting the interaction between HOTAIR and PRC2, thereby upregulating ZO-1 expression, which restricts RCC cell migration and invasion [45]. Additionally, genistein epigenetically activates the tumor suppressor gene BTG3. In RCC, BTG3 is frequently silenced due to the hypermethylation of its promoter region, leading to the loss of its function in inhibiting malignant tumor cell proliferation. Genistein significantly reduces the methylation levels of the BTG3 promoter and promotes histone H3 and H4 acetylation, which reactivates BTG3 expression, induces G1-phase cell cycle arrest, inhibits RCC cell proliferation, and promotes apoptosis [46]. Moreover, in both in vitro and in vivo experiments, genistein exhibits significant anti-angiogenic effects. It suppresses the expression of VEGF and basic fibroblast growth factor (bFGF), thereby inhibiting angiogenesis and reducing the blood supply to the tumor, which limits RCC growth and metastasis [47].

Collectively, isoflavones exhibit potent anti-RCC properties by inducing apoptosis, suppressing angiogenesis, altering cancer metabolism, and modulating epigenetic and miRNA-mediated pathways. In addition to their therapeutic effects, the cancer-preventive potential of isoflavones has also attracted growing attention [87]. Whether certain isoflavones can serve as chemopreventive agents against RCC remains an open and promising area of investigation. Such an approach may hold significant clinical value in reducing the incidence of RCC.

### 3.5. Chalcones

Chalcones are open-chain flavonoids with a characteristic α, β-unsaturated carbonyl system, known for their strong cytotoxic and pro-apoptotic effects against cancer cells. In RCC, chalcones have shown potential in triggering apoptosis and autophagy.

Isoliquiritigenin, a natural chalcone derived from *Glycyrrhiza uralensis* Fisch., exhibits pharmacological activities, including antioxidant, anti-inflammatory, and anticancer properties [88]. Kim et al. [48] found that isoliquiritigenin significantly reduces cell viability and induces apoptosis in RCC cells. Additionally, isoliquiritigenin decreases the expression of Bcl-2 and Bcl-xl, while increasing the expression of BAX, leading to cytochrome c release, generating ROS and activation of the mitochondrial pathway of apoptosis. Moreover, they confirmed that isoliquiritigenin inhibits the JAK2/STAT3 signaling pathway by decreasing p-JAK2 and p-STAT3, as well as the expression of STAT3 target genes, including cyclin D1 and D2.

Licochalcone A is a flavonoid natural product derived from the root of *Glycyrrhiza uralensis* Fisch. Currently, licochalcone A has been shown to possess pharmacological activities, including antibacterial, anti-inflammatory, antiviral, and anticancer effects [89]. In the context of RCC, Xin et al. [49] demonstrated that licochalcone A significantly inhibits the proliferation, migration, and invasion of RCC cells. Furthermore, licochalcone A significantly upregulates the expression of key autophagy-related proteins, including LC3-II, Beclin 1, and Atg5, while downregulating p62, supporting the role of licochalcone A in autophagy induction. Additionally, licochalcone A suppresses activation of the PI3K/Akt/mTOR signaling pathway. The use of the PI3K/Akt inhibitor (LY294002) and mTOR inhibitor (rapamycin) further confirmed that licochalcone A induces autophagy via inhibition of the PI3K/Akt/mTOR pathway.

In conclusion, chalcones act on RCC cells by inducing mitochondrial-dependent apoptosis, promoting autophagy, and inhibiting critical oncogenic pathways such as JAK2/STAT3 and PI3K/Akt/mTOR. Both apoptosis and autophagy are closely linked to mitochondrial function, and targeting mitochondria-mediated metabolic pathways has emerged as a key strategy in RCC therapy [90]. Future studies integrating metabolomics approaches to investigate whether chalcones exert anti-RCC effects through metabolic regulation will provide deeper insights into their anticancer mechanisms and therapeutic potential.

### 3.6. Flavanols

Flavanols, particularly epigallocatechin gallate (EGCG) from green tea, have been extensively studied for their anticancer effects.

Epigallocatechin gallate (EGCG) is a flavanol extracted from green tea [91]. The antitumor effects of EGCG have garnered significant research interest, and its mechanisms of action in RCC have also been investigated. Previous studies [50,51] have shown that EGCG upregulates the expression of tissue factor pathway inhibitor-2 (TFPI-2), a factor negatively correlated with tumor malignancy. This modulation results in the inhibition of cell proliferation, migration, invasion, and the EMT process. Additionally, EGCG has been found to enhance TRAIL-induced apoptosis in RCC cells. More recently, Liu et al. [52] demonstrated that EGCG upregulates the expression of transcription factor EB (TFEB), thereby activating autophagy.

Although current research on flavanols in RCC remains limited, several flavanols, such as abacopterin C, eruberin C, and triphyllin A, have demonstrated antitumor effects in other cancer types [92]. These findings suggest a promising therapeutic potential that has yet to be fully explored in the context of RCC. Future studies are needed to further investigate the anti-RCC properties of flavanols and expand their application in RCC therapy.

## 4. Future Research Directions

Despite extensive studies highlighting the potential of flavonoids in the treatment of RCC, most of these investigations remain at the preclinical stage. To facilitate the clinical translation of flavonoids and optimize their therapeutic efficacy in RCC, further in-depth research is essential. Future research on flavonoids in RCC treatment will primarily focus on improving their bioavailability to optimize their therapeutic effects and exploring combination therapies to augment the efficacy of existing RCC treatments.

### 4.1. Enhancing the Bioavailability of Flavonoids

One of the major challenges in translating the promising anticancer potential of flavonoids into effective clinical therapies for RCC is their poor bioavailability. Flavonoids, despite their potent therapeutic properties, often exhibit limited absorption, rapid metabolism, and quick elimination from the body, which significantly reduces their efficacy. The low water solubility, poor gastrointestinal stability, and extensive first-pass metabolism are key factors contributing to the poor bioavailability of flavonoids. To overcome these challenges and maximize the therapeutic potential of flavonoids in RCC treatment, researchers are exploring various strategies to enhance their bioavailability. These strategies can be broadly classified into the synthesis of novel flavonoid derivatives and the use of drug delivery systems [93].

#### 4.1.1. Novel Flavonoid Derivatives

The development of natural product derivatives, particularly flavonoid-based compounds, offers a promising strategy for enhancing the stability, bioavailability, and therapeutic efficacy of antitumor agents. These derivatives are synthesized by modifying the chemical structure of natural products, thus optimizing their pharmacological properties in a cost-effective and practical manner. Recent advances highlight the potential of such derivatives in RCC treatment, a field where novel, more effective therapies are urgently needed.

In a study conducted by Li et al. [94], the peripheral substituents of apigenin—a well-known flavonoid with anticancer properties—were modified to create a series of 11 derivatives, labeled 15a–k. Among these, derivative 15e, which incorporates a *para*-acetophenyl nonpolar group, demonstrated a remarkable improvement in therapeutic efficacy. The IC_50_ value of 15e against RCC cells was 100 times lower than that of unmodified apigenin, showcasing a substantial enhancement in potency. Mechanistic studies further revealed that 15e targets the MET protein, a key player in RCC progression [95]. Importantly, 15e also exhibited activity against MET mutant RCC cells that are resistant to conventional therapies, including crizotinib and cabozantinib. This characteristic is highly significant, as drug resistance remains a major barrier to the effective treatment of RCC. The promising results obtained with apigenin derivative 15e underscore the potential of structure-optimized flavonoid derivatives in RCC therapy. Moving forward, future research could focus on further optimization of flavonoid derivatives to improve selectivity and reduce potential side effects.

By exploring the potential of novel flavonoid derivatives, researchers can address some of the most pressing challenges in terms of RCC treatment, such as drug resistance and low bioavailability, thereby advancing the clinical utility of flavonoid-based therapies.

#### 4.1.2. Drug Delivery Systems

**Figure 1:**
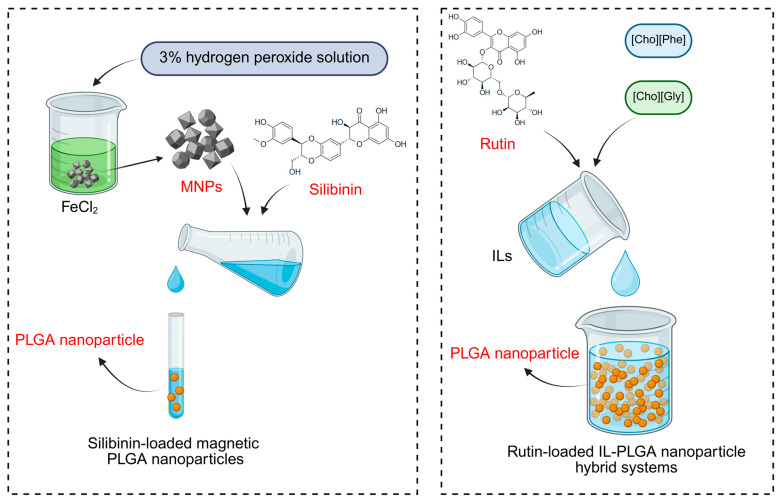
Combining flavonoids with nanomaterials for treatment of RCC.

Combining natural products, such as flavonoids, with advanced drug delivery technologies, such as nanoformulation, liposomal encapsulation, and bioenhancers, can significantly enhance their pharmacokinetic properties, making them more effective in cancer treatment [96]. Research has increasingly shown that nanomaterials represent a promising avenue for enhancing the anticancer potential of flavonoids against RCC (Figure 1).

For example, silibinin, a flavonoid derived from *Silybum marianum* (L.) Gaertn., has shown anticancer effects against RCC in both in vivo and in vitro studies. Silibinin’s therapeutic effects are achieved through mechanisms including the inhibition of EMT, induction of apoptosis, and promotion of autophagy [97]. However, when Takke et al. [98] loaded silibinin onto magnetic PLGA nanoparticles (MPNPs), they observed a notable enhancement of drug performance. The silibinin-loaded magnetic PLGA nanoparticles (SLB-MPNPs) exhibited minimal cytotoxicity, increased stability, prolonged action time, and improved therapeutic efficacy in treating RCC compared to silibinin alone. In a related development, Caparica et al. [99] designed an ionic liquid (IL)–nanoparticle hybrid delivery system for rutin, another plant-derived flavonoid. The IL–nanoparticle hybrid not only preserved the activity of rutin but also improved its solubility and extended its drug release time, addressing two major barriers to flavonoid efficacy.

Future research should continue to explore and optimize these delivery systems, focusing on materials that offer high biocompatibility, minimal cytotoxicity, and precise targeting of RCC cells. Developing such advanced systems can further improve the therapeutic efficacy and safety profile of flavonoid-based treatments for RCC, potentially leading to more effective and less invasive therapeutic options for patients.

### 4.2. Combination Therapy Strategies

The low toxicity and multifunctional properties of natural products make them highly attractive candidates for adjunctive therapies in cancer treatment. Combining natural products such as flavonoids with conventional therapies offers an innovative approach to enhance the effectiveness of existing treatments, mitigate adverse side effects, and address the problem of treatment resistance that often complicates RCC management [100]. Recent studies have underscored the potential of several flavonoids to augment RCC treatment efficacy, particularly when combined with conventional therapies such as TKIs, ICIs, and chemotherapy.

For instance, wogonin, a flavonoid derived from *Scutellaria baicalensis* Georgi, has demonstrated both standalone anticancer effects and the ability to overcome drug resistance in RCC. Wogonin achieves this by inhibiting the CDK4-RB pathway, which regulates cell cycle progression, thereby enhancing the efficacy of sunitinib. This synergistic action addresses the challenge of resistance to targeted therapies in RCC [101]. Similarly, cyanidin, an anthocyanin present in various fruits and vegetables, has shown notable anticancer activity in RCC. Liu et al. [102] demonstrated that cyanidin significantly inhibits RCC cell proliferation and migration in vitro and in vivo. Furthermore, cyanidin was shown to induce apoptosis by upregulating EGFR1 expression and downregulating SEPW1 expression. Additionally, the combination of cyanidin with cisplatin has been found to increase RCC cells’ sensitivity to cisplatin, further supporting the potential of flavonoid-based combination therapies in RCC treatment.

Fisetin, a flavonol found in fruits such as strawberries, has garnered attention as a potential enhancer of conventional RCC therapies. Initially, fisetin was shown to inhibit RCC cell proliferation and migration through activation of the MEK/ERK pathway, resulting in the downregulation of CTSS and ADAM9 expression [103]. Subsequent research further validated fisetin’s potential to enhance chemotherapy efficacy in RCC when used in combination therapies. Jiang et al. [104] showed that fisetin, when combined with cisplatin, significantly improved the inhibitory effect of cisplatin on RCC cells compared to cisplatin alone. This enhancement was attributed to fisetin’s ability to sensitize RCC cells to cisplatin by targeting the PI3K/Akt/mTOR signaling pathway. Moreover, fisetin has been found to increase RCC cells’ sensitivity to sunitinib, suggesting that it could reduce the required doses of standard treatments, thereby lowering the risk of toxic side effects.

Tangeretin, a flavonoid derived from citrus peel, has shown potential in overcoming drug resistance in various cancers, including colorectal and breast cancer [105]. In the context of RCC, tangeretin has been found to target connexin43 (Cx43), a biomarker associated with a poor prognosis in RCC. By modulating Cx43, tangeretin inhibits RCC cell viability and migration, while also enhancing RCC cells’ sensitivity to TKIs such as sunitinib and sorafenib [106].

For the successful integration of flavonoid-based adjunctive therapies into RCC treatment regimens, further investigation is needed. Key areas of focus should include determining the optimal dosing, timing, and sequencing of flavonoid combinations with existing therapies, as well as understanding the precise mechanisms through which flavonoids enhance treatment efficacy. Clinical trials will be essential to evaluate the safety and effectiveness of flavonoid-based combination therapies in RCC patients, paving the way for more personalized, effective, and less toxic treatment strategies in the future.

## 5. Conclusions

To our knowledge, this review offers a novel and in-depth analysis of flavonoids as potential anti-RCC agents, with unique insights into their mechanisms of action, molecular targets, and clinical implications for future strategies. By presenting these perspectives, we aim to enhance understanding of flavonoid-based therapies and facilitate their clinical translation, proposing innovative approaches to improve RCC treatment outcomes.

**Figure 2:**
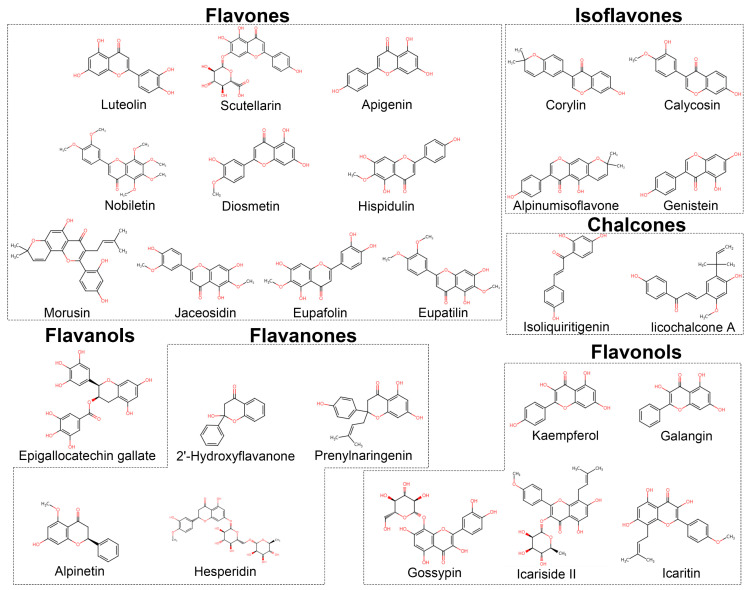
Chemical structures of flavonoids for treatment of RCC.

**Figure 3:**
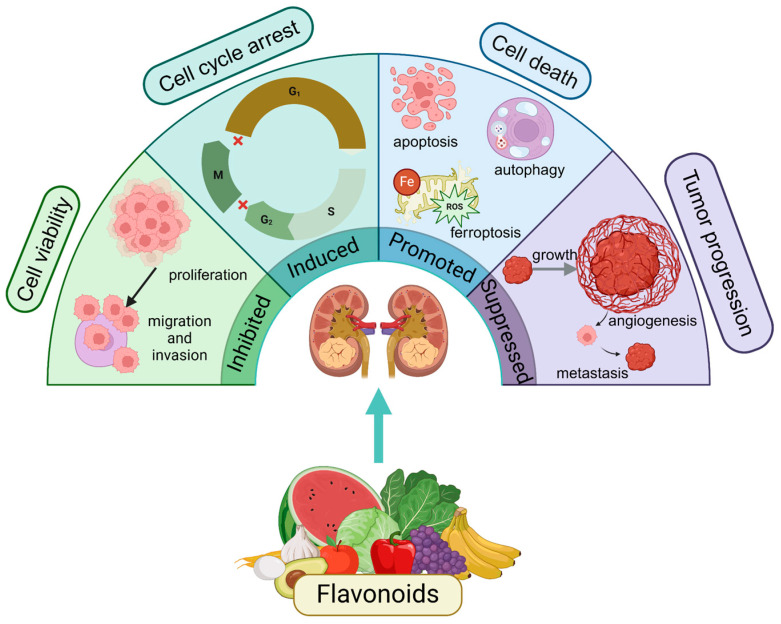
Mechanisms of flavonoids against RCC.

This review provides a comprehensive analysis of the anti-RCC effects of 26 natural flavonoids, categorized into 10 flavones, four flavanones, five flavonols, four isoflavones, two chalcones and one flavanol (Figure 2). By mapping these bioactivities onto the genomic landscape of RCC, we provide a systematical understanding of the potential therapeutic mechanisms underlying their effects. This review also highlights the multifaceted modes of action of flavonoids, including inhibition of RCC cell proliferation, migration, and invasion, induction of cell cycle arrest, and promotion of various forms of programmed cell death, such as apoptosis, autophagy, and ferroptosis. Furthermore, these compounds demonstrate notable efficacy in suppressing tumor growth, angiogenesis, and metastasis in RCC models, underscoring their diverse antitumor properties (Figure 3). Notably, this review emphasizes the ability of flavonoids to target critical signaling pathways involved in RCC progression and therapeutic resistance, such as JAK/STAT3, EGFR/PI3K/Akt/mTOR, and CaMKKβ/AMPK/USP51. In addition, this review uniquely contributes by exploring the miRNA-regulated mechanisms, including the miR-21/YAP1, miR-324-3p/GPX4, and miR-101/RLIP76 axes, which play a pivotal role in flavonoid-mediated anti-RCC actions. This perspective opens up new avenues for targeted modulation of specific molecular vulnerabilities in RCC, thus representing a significant advancement in precision medicine for RCC.

While flavonoids show great promise, we also address the challenges that remain in relation to their clinical application, such as limited bioavailability, metabolic instability, and potential off-target effects. Advances in nanotechnology and drug delivery systems could address these limitations by enhancing flavonoid stability, targeted delivery, and tissue penetration. Moreover, the integration of flavonoids into combination therapies, alongside conventional chemotherapeutics or immunotherapy agents, holds significant promise for synergistic effects and overcoming drug resistance in RCC.

Notably, although flavonoids exhibit promising medicinal value, excessive intake may pose certain toxicity risks. High doses of flavonoids can potentially promote oxidative stress, increase the metabolic burden on the liver, and interact with other drugs [107]. For instance, high doses of naringenin have been reported to induce oxidative stress in the testes by promoting ROS production and lipid peroxidation, which subsequently affects the sperm count and motility [108]. Similarly, diethyldithiocarbamate (DEDTC), a promising anticancer agent, when combined with EGCG, has been found to reduce EGCG’s cytotoxic effects while inducing oxidative stress and DNA damage in the liver, ultimately leading to hepatotoxicity [109]. Therefore, to ensure the safe clinical application of flavonoids in the treatment of RCC and other diseases, it is crucial to explore appropriate dosage ranges and optimal combination therapy strategies.

Currently, several clinical trials are underway to evaluate the feasibility of flavonoids in the treatment of various diseases, such as gastrointestinal inflammation, leukemia, and ovarian cancer [110,111]. A phase II clinical trial investigated the efficacy of flavopiridol in the treatment of advanced RCC. The results indicated that among 34 enrolled patients, 12% exhibited a positive response, with a median overall survival of 9 months [112]. Although the therapeutic efficacy was limited, this study provided clinical evidence supporting the biological activity of flavopiridol in the treatment of advanced RCC. Looking ahead, to better realize the potential of flavonoids as anticancer agents in the clinical management of RCC, further clinical trials are essential to validate their efficacy and optimize their therapeutic application.

In conclusion, flavonoids represent a highly promising class of natural compounds with the potential to revolutionize RCC treatment. Future research should focus on elucidating their molecular targets, optimizing their clinical application, and validating their efficacy in clinical trials. With continued scientific exploration and innovation, flavonoids may offer transformative, personalized, and minimally invasive treatment options, improving both the survival outcomes and the quality of life of patients with RCC.

## Figures and Tables

**Table 1:** List of flavonoids used as therapy against RCC.

Categories	Name	Mechanism	Model		References
			Cell Lines Tested	Animal Models Used	
Flavones	Luteolin	1. Induced ferroptosis by promoting heme degradation and iron accumulation through upregulation of HO-1 expression. 2. Enhanced TRAIL-induced apoptosis by downregulating FLIP.	RCC cell lines: 786-O (10–40 μM), OS-RC-2 (60 μM), ACHN (10 μM) and A498 (10 μM)	/	[16,17]
	Scutellarin	1. Inhibited cell proliferation and invasion by reducing MMP-2 and MMP-9. 2. Induced G0/G1 phase cell cycle arrest and promoted apoptosis. 3. Targeted PI3K/Akt/mTOR pathway and upregulated PTEN protein levels.	RCC cell lines: ACHN and 786-O (30–90 μM)	/	[18]
	Apigenin	1. Inhibited cell proliferation by inducing DNA damage and G2/M phase cell cycle arrest. 2. Regulated cell cycle progression by downregulating the cyclins and CDK1.	1. RCC cell lines: ACHN (10–50 μM), Caki-1 (20–50 μM), 786-O (10–50 μM) and NC65 (0–100 μM) 2. Primary RCC cells (20–50 μM)	/	[19,20]
	Nobiletin	1. Reversed hypoxia-induced EMT process in RCC cells. 2. Targeted the SRC/AKT pathway, JAK2/STAT3 pathway, and PI3K/Akt pathway. 3. Inhibited cell proliferation, migration, and invasion, while inducing apoptosis. 4. Targeted SKP2 to enhances RCC sensitivity to palbociclib.	RCC cell lines: ACHN (80–120 μM), Caki-2 (40–80 μM), 786-O (12.5–100 μM), Caki-1 (12.5–25 μM), 769-P (12.5–25 μM) and OSRC-2 (25–50 μM)	Xenograft RCC nude mice (40 mg/kg)	[21,22,23,24]
	Diosmetin	1. Inhibited cell viability and induced apoptosis. 2. Disrupted the PI3K/Akt pathway by inducing p53 activation.	RCC cell lines: Caki-1 (5–20 μM)	/	[25]
	Hispidulin	Enhanced TRAIL-induced apoptosis by regulating CaMKKβ/AMPK/USP51 signaling pathway.	RCC cell lines: ACHN, Caki-1, A498 and DU145 (10–30 μM)	Xenograft RCC nude mice (10 mg/kg)	[26]
	Morusin	1. Inhibited cell proliferation and invasion. 2. Induced cell cycle arrest and promoted apoptosis by targeting MAPK pathway.	RCC cell lines: 769-P, 786-O and OSRC-2 (2–8 μg/mL)	Xenograft RCC nude mice (20 mg/kg)	[27]
	Jaceosidin	1. Disrupted mitochondrial integrity. 2. Induced apoptosis by activating BAX and downregulating Mcl-1.	RCC cell lines: Caki-1, ACHN, A498 and 786-O (30–75 μM)	/	[28]
	Eupafolin	Enhanced TRAIL-induced apoptosis by modulating Mcl-1 and Bim expression.	RCC cell lines: Caki-1 (10–30 μM)	Xenograft RCC nude mice (10 mg/kg)	[29]
	Eupatilin	1. Inhibited cell proliferation and migration. 2. Induced apoptosis by regulating miR-21/YAP1 axis.	RCC cell lines: 786-O (5–20 μM)	Xenograft RCC nude mice (10 mg/kg)	[30]
Flavanones	Alpinetin	1. Inhibited cell proliferation and migration, and promoted apoptosis. 2. Targeted the PI3K/AKT/mTOR pathway.	RCC cell lines: 786-O and OS-RC-2 (50–100 μM)	Xenograft RCC nude mice (100 mg/kg)	[31]
	Hesperidin	1. Inhibited RCC progression by targeting the COX-2/PGE2 pathway. 2. Prevented RCC by enhancing the renal antioxidant defense system.	/	Diethylnitrosamine initiated and ferric nitrilotriacetate promoted renal carcinogenesis in Wistar rats (100 or 200 mg/kg)	[32]
	2′-Hydroxyflavanone	1. Suppressed cell division by inducing G2/M cell cycle arrest. 2. Inhibited VHL-mutant RCC growth and metastasis by reducing EGFR/PI3K/Akt signaling pathways. 3. Promoted oxidative stress in RCC cells. 4. Reduced angiogenesis by lowering VEGF.	RCC cell lines: Caki-1 (20–100 μM), Caki-2 (25–50 μM), A498 (20–100 μM) and 786-O (25–50 μM)	Xenograft RCC nude mice (25 or 100 mg/kg)	[33]
	Prenylnaringenin	Inhibited cell proliferation.	human renal carcinoma cell lines: UO.31 (6.25–100 μM)	/	[34]
Flavonols	Kaempferol	1. Induced cell cycle arrest and apoptosis by targeting the EGFR/p38 pathway. 2. Inhibited cell invasion and migration by targeting the AKT and FAK pathways.	RCC cell lines: 769-P (50–150 μM) and 786-O (25–150 μM)	Xenograft RCC severe combined immunodeficient mice (2 or 10 mg/kg)	[35,36]
	Galangin	1. Induced TRAIL-mediated apoptosis by inhibiting Bcl-2 and Mcl-1. 2. Inhibited cell proliferation and migration, and induced apoptosis by increasing intracellular ROS levels.	RCC cell lines: 786-O (25–100 μM), Caki-1 (10–100 μM), ACHN (10–30 μM) and A498 (10–30 μM)	/	[37,38]
	Icariside II	1. Inhibited cell viability, proliferation, and migration. 2. Induced ferroptosis by regulating miR-324-3p/GPX4 axis.	RCC cell lines: ACHN and Caki-1 (10–40 μM)	Xenograft RCC nude mice (15 or 25 or 35 mg/kg)	[39]
	Icaritin	1. Inhibited cell proliferation and induced apoptosis by targeting the JAK/STAT3 pathway. 2. Suppressed tumor growth and inhibited angiogenesis.	RCC cell lines: 786-O (1–30 μM)	Xenograft RCC BALB/c mice (10 mg/kg)	[40]
	Gossypin	Inhibited tumor growth by reducing NFκB and STAT3, and targeting PI3K/Akt/mTOR signaling pathway.	/	Xenograft RCC nude mice	[41]
Isoflavones	Corylin	1. Inhibited cell proliferation by blocking cell cycle. 2. Induced apoptosis and suppressed cell migration and invasion. 3. Suppressed energy metabolism by downregulating RAGE.	RCC cell lines: 786-O and A498 (5–40 μM)	Xenograft RCC NSG BALB/c mice (30 or 60 mg/kg)	[42]
	Calycosin	1. Suppressed cell proliferation, migration, and invasion. 2. Promoted apoptosis by targeting MAZ/HAS2 signaling pathway.	RCC cell lines: 786-O (120–180 μM) and A498 (90–120 μM)	Xenograft RCC nude mice (40 mg/kg)	[43]
	Alpinumisoflavone	Inhibited tumor growth and metastasis through modulating miR-101/RLIP76 signaling pathway.	RCC cell lines: 786-O (2.5–10 μM)	Xenograft RCC NSG BALB/c mice (40 or 80 mg/kg)	[44]
	Genistein	1. Inhibited cell migration and invasion by disrupting the binding of HOTAIR with PRC2 and upregulating ZO-1 expression. 2. Induced cell cycle arrest and inhibited cell proliferation by activating the BTG3 through demethylation and histone modifications. 3. Restricted tumor growth and dissemination by suppressing VEGF and bFGF expression and angiogenesis.	RCC cell lines: SMKT R1-4 (12.5–100 μg/mL), A498 (50 μM), HEK-293 (50 μM), 786-O (25 μM) and ACHN (25–50 μM)	/	[45,46,47]
Chalcones	Isoliquiritigenin	1. Inhibitied RCC cells viability. 2. Induced apoptosis by decreasing Bcl-2 and Bcl-xl, increasing BAX and generating ROS. 3. Targeted the JAK2/STAT3 signaling pathway.	RCC cell lines: Caki-1 (5–50 μM)	/	[48]
	Licochalcone A	1. Induced autophagy and suppressed proliferation, migration, and invasion. 2. Inhibited the PI3K/Akt/mTOR signaling pathway.	RCC cell lines: 786-O and 769-P (10–50 μM)	/	[49]
Flavanols	Epigallocatechin gallate	1. Inhibited cell proliferation, migration, invasion, and the EMT process by upregulating TFPI-2. 2. Enhanced TRAIL-induced apoptosis. 3. Activated autophagy by upregulating TFEB.	RCC cell lines: 786-O (50 μg/mL or 10–80 μM) and ACHN (20–80 μM)	Xenograft RCC nude mice (50 mg/kg)	[50,51,52]

Note: RCC: Renal cell carcinoma; HO-1: Heme oxygenase-1; TRAIL: Tumor necrosis factor-related apoptosis-inducing ligand; ROS: Reactive oxygen species; EMT: Epithelial–mesenchymal transition; TFPI-2: Tissue factor pathway inhibitor-2; TFEB: Transcription factor EB; VEGF: Vascular endothelial growth factor; RAGE: Receptor for advanced glycation end products; bFGF: Basic fibroblast growth factor.

## Data Availability

All new data have been presented in this paper. There are no further data, but the authors welcome questions and discussion.

## Abbreviations

The following abbreviations are used in this manuscript:

RCC	Renal cell carcinoma
ICIs	Immune checkpoint inhibitors
TKIs	Tyrosine kinase inhibitors
OS	Overall survival
PFS	Progression-free survival
TRAIL	Tumor necrosis factor-related apoptosis-inducing ligand
HO-1	Heme oxygenase-1
ROS	Reactive oxygen species
EMT	Epithelial–mesenchymal transition
EGCG	Epigallocatechin gallate
TFPI-2	Tissue factor pathway inhibitor-2
TFEB	Transcription factor EB
VEGF	Vascular endothelial growth factor
GSTp	Glutathione S-transferase pi
6-PN	6-Prenylnaringenin
8-PN	8-Prenylnaringenin
SOD	Superoxide dismutase
MDA	Malondialdehyde
RAGE	Receptor for advanced glycation end products
HA	Hyaluronic acid
ECM	Extracellular matrix
bFGF	Basic fibroblast growth factor
MPNPs	Magnetic PLGA nanoparticles
SLB-MPNPs	Silibinin-loaded magnetic PLGA nanoparticles
Cx43	Connexin43
DEDTC	Diethyldithiocarbamate

## References

[1] Bray F., Laversanne M., Sung H., Ferlay J., Siegel R.L., Soerjomataram I., Jemal A. (2024). Global cancer statistics 2022: GLOBOCAN estimates of incidence and mortality worldwide for 36 cancers in 185 countries. CA Cancer J. Clin..

[2] Evans S.T., Jani Y., Jansen C.S., Yildirim A., Kalemoglu E., Bilen M.A. (2024). Understanding and overcoming resistance to immunotherapy in genitourinary cancers. Cancer Biol. Ther..

[3] Numakura K., Sekine Y., Hatakeyama S., Muto Y., Sobu R., Kobayashi M., Sasagawa H., Kashima S., Yamamto R., Nara T. (2023). Primary resistance to nivolumab plus ipilimumab therapy in patients with metastatic renal cell carcinoma. Cancer Med..

[4] Huang T., Peng Y., Liu R., Ma B., Chen J., Wei W., Zhong W., Liu Y., Guo S., Han H. (2024). Prognostic significance of immune evasion-related genes in clear cell renal cell carcinoma immunotherapy. Int. Immunopharmacol..

[5] Lee S.H. (2024). Therapeutic Effects of Natural Products on Human Diseases. Life.

[6] Dastmalchi M., Chen X., Hagel J.M., Chang L., Chen R., Ramasamy S., Yeaman S., Facchini P.J. (2019). Neopinone isomerase is involved in codeine and morphine biosynthesis in opium poppy. Nat. Chem. Biol..

[7] Koehn F.E., Carter G.T. (2005). The evolving role of natural products in drug discovery. Nat. Rev. Drug Discov..

[8] Ghosh S., Das S.K., Sinha K., Ghosh B., Sen K., Ghosh N., Sil P.C. (2024). The Emerging Role of Natural Products in Cancer Treatment. Arch. Toxicol..

[9] Newman D.J., Cragg G.M. (2020). Natural Products as Sources of New Drugs over the Nearly Four Decades from 01/1981 to 09/2019. J. Nat. Prod..

[10] Chen S., Wang X., Cheng Y., Gao H., Chen X. (2023). A Review of Classification, Biosynthesis, Biological Activities and Potential Applications of Flavonoids. Molecules.

[11] Das A., Ruhal R. (2025). Potential of plants-based alkaloids, terpenoids and flavonoids as antibacterial agents: An update. Process Biochem..

[12] Alizadeh A., Pourfallah-Taft Y., Khoshnazar M., Safdari A., Komari S.V., Zanganeh M., Sami N., Valizadeh M., Faridzadeh A., Alijanzadeh D. (2024). Flavonoids against depression: A comprehensive review of literature. Front. Pharmacol..

[13] Vazhappilly C.G., Amararathna M., Cyril A.C., Linger R., Matar R., Merheb M., Ramadan W.S., Radhakrishnan R., Rupasinghe H.P.V. (2021). Current methodologies to refine bioavailability, delivery, and therapeutic efficacy of plant flavonoids in cancer treatment. J. Nutr. Biochem..

[14] Tiwari P., Mishra K.P. (2023). Role of Plant-Derived Flavonoids in Cancer Treatment. Nutr. Cancer.

[15] Kustiawan P.M., Siregar K.A.A.K., Jauhar M.M., Ramadhan D., Mardliyati E., Syaifie P.H. (2024). Network pharmacology and bioinformatic integrative analysis reveals candidate gene targets and potential therapeutic of East Kalimantan propolis against hepatocellular carcinoma. Heliyon.

[16] Han S., Lin F., Qi Y., Liu C., Zhou L., Xia Y., Chen K., Xing J., Liu Z., Yu W. (2022). HO-1 Contributes to Luteolin-Triggered Ferroptosis in Clear Cell Renal Cell Carcinoma via Increasing the Labile Iron Pool and Promoting Lipid Peroxidation. Oxidative Med. Cell. Longev..

[17] Ou Y.C., Li J.R., Kuan Y.H., Raung S.L., Wang C.C., Hung Y.Y., Pan P.H., Lu H.C., Chen C.J. (2014). Luteolin sensitizes human 786-O renal cell carcinoma cells to TRAIL-induced apoptosis. Life Sci..

[18] Deng W., Han W., Fan T., Wang X., Cheng Z., Wan B., Chen J. (2018). Scutellarin inhibits human renal cancer cell proliferation and migration via upregulation of PTEN. Biomed. Pharmacother..

[19] Meng S., Zhu Y., Li J.F., Wang X., Liang Z., Li S.Q., Xu X., Chen H., Liu B., Zheng X.Y. (2017). Apigenin inhibits renal cell carcinoma cell proliferation. Oncotarget.

[20] Bao Y., Wu X., Jin X., Kanematsu A., Nojima M., Kakehi Y., Yamamoto S. (2022). Apigenin inhibits renal cell carcinoma cell proliferation through G2/M phase cell cycle arrest. Oncol. Rep..

[21] Liu F., Zhang S., Yin M., Guo L., Xu M., Wang Y. (2019). Nobiletin inhibits hypoxia-induced epithelial-mesenchymal transition in renal cell carcinoma cells. J. Cell Biochem..

[22] Wei D., Zhang G., Zhu Z., Zheng Y., Yan F., Pan C., Wang Z., Li X., Wang F., Meng P. (2019). Nobiletin Inhibits Cell Viability via the SRC/AKT/STAT3/YY1AP1 Pathway in Human Renal Carcinoma Cells. Front. Pharmacol..

[23] Xu Z., Wu D., Fu D., Tang C., Ge J., Zhang Z., Zhou W. (2020). Nobiletin inhibits viability of human renal carcinoma cells via the JAK2/STAT3 and PI3K/Akt pathway. Cell. Mol. Biol..

[24] Chen T., Liu L., Zou Y., Hu X., Zhang W., Zhou T., Luo X., Fu W., Xu J. (2021). Nobiletin downregulates the SKP2-p21/p27-CDK2 axis to inhibit tumor progression and shows synergistic effects with palbociclib on renal cell carcinoma. Cancer Biol. Med..

[25] Sun Z., Liu K., Liang C., Wen L., Wu J., Liu X., Li X. (2024). Diosmetin as a promising natural therapeutic agent: In vivo, in vitro mechanisms, and clinical studies. Phytother. Res..

[26] Woo S.M., Seo S.U., Kim S.H., Nam J.O., Kim S., Park J.W., Min K.J., Kwon T.K. (2019). Hispidulin Enhances TRAIL-Mediated Apoptosis via CaMKKβ/AMPK/USP51 Axis-Mediated Bim Stabilization. Cancers.

[27] Yang C., Luo J., Luo X., Jia W., Fang Z., Yi S., Li L. (2020). Morusin exerts anti-cancer activity in renal cell carcinoma by disturbing MAPK signaling pathways. Ann. Transl. Med..

[28] Woo S.M., Kwon T.K. (2016). Jaceosidin induces apoptosis through Bax activation and down-regulation of Mcl-1 and c-FLIP expression in human renal carcinoma Caki cells. Chem. Biol. Interact..

[29] Han M.A., Min K.J., Woo S.M., Seo B.R., Kwon T.K. (2016). Eupafolin enhances TRAIL-mediated apoptosis through cathepsin S-induced down-regulation of Mcl-1 expression and AMPK-mediated Bim up-regulation in renal carcinoma Caki cells. Oncotarget.

[30] Zhong W., Wu Z., Chen N., Zhong K., Lin Y., Jiang H., Wan P., Lu S., Yang L., Liu S. (2019). Eupatilin Inhibits Renal Cancer Growth by Downregulating MicroRNA-21 through the Activation of YAP1. BioMed Res. Int..

[31] Guo Y., Jiang L., Luo S., Hu D., Zhao X., Zhao G., Tang W. (2024). Network Analysis and Basic Experiments on the Inhibition of Renal Cancer Proliferation and Migration by Alpinetin through PI3K/AKT/mTOR Pathway. Curr. Mol. Med..

[32] Siddiqi A., Saidullah B., Sultana S. (2018). Anti-carcinogenic effect of hesperidin against renal cell carcinoma by targeting COX-2/PGE2 pathway in Wistar rats. Environ. Toxicol..

[33] Nagaprashantha L.D., Vatsyayan R., Singhal J., Lelsani P., Prokai L., Awasthi S., Singhal S.S. (2011). 2′-hydroxyflavanone inhibits proliferation, tumor vascularization and promotes normal differentiation in VHL-mutant renal cell carcinoma. Carcinogenesis.

[34] Busch C., Noor S., Leischner C., Burkard M., Lauer U.M., Venturelli S. (2015). Anti-proliferative activity of hop-derived prenylflavonoids against human cancer cell lines. Wien. Med. Wochenschr..

[35] Song W., Dang Q., Xu D., Chen Y., Zhu G., Wu K., Zeng J., Long Q., Wang X., He D. (2014). Kaempferol induces cell cycle arrest and apoptosis in renal cell carcinoma through EGFR/p38 signaling. Oncol. Rep..

[36] Hung T.W., Chen P.N., Wu H.C., Wu S.W., Tsai P.Y., Hsieh Y.S., Chang H.R. (2017). Kaempferol Inhibits the Invasion and Migration of Renal Cancer Cells through the Downregulation of AKT and FAK Pathways. Int. J. Med. Sci..

[37] Han M.A., Lee D.H., Woo S.M., Seo B.R., Min K.J., Kim S., Park J.W., Kim S.H., Choi Y.H., Kwon T.K. (2016). Galangin sensitizes TRAIL-induced apoptosis through down-regulation of anti-apoptotic proteins in renal carcinoma Caki cells. Sci. Rep..

[38] Cao J., Wang H., Chen F., Fang J., Xu A., Xi W., Zhang S., Wu G., Wang Z. (2016). Galangin inhibits cell invasion by suppressing the epithelial-mesenchymal transition and inducing apoptosis in renal cell carcinoma. Mol. Med. Rep..

[39] Yu R., Zhou Y., Shi S., Wang X., Huang S., Ren Y. (2022). Icariside II induces ferroptosis in renal cell carcinoma cells by regulating the miR-324-3p/GPX4 axis. Phytomedicine.

[40] Li S., Priceman S.J., Xin H., Zhang W., Deng J., Liu Y., Huang J., Zhu W., Chen M., Hu W. (2013). Icaritin inhibits JAK/STAT3 signaling and growth of renal cell carcinoma. PLoS ONE.

[41] Li S., An F., Guo J.Y., Gu D.Q., Zhao C.L., Li Y., Ji J. (2023). Gossypin Inhibits Renal Cell Carcinoma Growth In Vivo through Targeting the Inflammatory Reaction. Lat. Am. J. Pharm..

[42] Yang M., Wu X., Liu N., Hu X., Shi X. (2024). The isoflavone corylin suppresses tumor growth and metastasis in renal cell carcinoma by regulating RAGE transcription. J. Funct. Foods.

[43] Zhang Z.H., Yuan C.Y., Xu M., Wang M.F., Feng T., Wang Y., Zheng S.F., Zhang H.L., Shi G.H., Cao D.L. (2024). Calycosin inhibits the proliferation and metastasis of renal cell carcinoma through the MAZ/HAS2 signaling pathway. Phytother. Res..

[44] Wang T., Jiang Y., Chu L., Wu T., You J. (2017). Alpinumisoflavone suppresses tumour growth and metastasis of clear-cell renal cell carcinoma. Am. J. Cancer Res..

[45] Imai-Sumida M., Dasgupta P., Kulkarni P., Shiina M., Hashimoto Y., Shahryari V., Majid S., Tanaka Y., Dahiya R., Yamamura S. (2020). Genistein Represses HOTAIR/Chromatin Remodeling Pathways to Suppress Kidney Cancer. Cell Physiol. Biochem..

[46] Majid S., Dar A.A., Ahmad A.E., Hirata H., Kawakami K., Shahryari V., Saini S., Tanaka Y., Dahiya A.V., Khatri G. (2009). BTG3 tumor suppressor gene promoter demethylation, histone modification and cell cycle arrest by genistein in renal cancer. Carcinogenesis.

[47] Sasamura H., Takahashi A., Yuan J., Kitamura H., Masumori N., Miyao N., Itoh N., Tsukamoto T. (2004). Antiproliferative and antiangiogenic activities of genistein in human renal cell carcinoma. Urology.

[48] Kim D.H., Park J.E., Chae I.G., Park G., Lee S., Chun K.S. (2017). Isoliquiritigenin inhibits the proliferation of human renal carcinoma Caki cells through the ROS-mediated regulation of the Jak2/STAT3 pathway. Oncol. Rep..

[49] Xin H., Xu W. (2018). Effect of licochalcone A on autophagy in renal cell carcinoma via PI3K/Akt/mTOR signaling pathway. Zhongguo Zhong Yao Za Zhi.

[50] Gu B., Ding Q., Xia G., Fang Z. (2009). EGCG inhibits growth and induces apoptosis in renal cell carcinoma through TFPI-2 overexpression. Oncol. Rep..

[51] Wei R., Zhu G., Jia N., Yang W. (2015). Epigallocatechin-3-gallate Sensitizes Human 786-O Renal Cell Carcinoma Cells to TRAIL-Induced Apoptosis. Cell Biochem. Biophys..

[52] Liu B., Luo L., Yu B., Que T., Zhang Y. (2024). EGCG inhibits migration, invasion and epithelial-mesenchymal transition of renal cell carcinoma by activating TFEB-mediated autophagy. Chem. Biol. Interact..

[53] Zhang W., Zhang F. (2024). Exploration of the mechanism of luteolin against colorectal cancer based on network pharmacology and experimental validation. Asian J. Surg..

[54] Nguyen T.T.K., Woo S.M., Seo S.U., Banstola A., Kim H., Duwa R., Vu A.T.T., Hong I.S., Kwon T.K., Yook S. (2025). Enhanced anticancer efficacy of TRAIL-conjugated and odanacatib-loaded PLGA nanoparticles in TRAIL resistant cancer. Biomaterials.

[55] Qiao L., Liu K., Ren Y., Liu Y., Xu Z., Wang S., Zhang Y. (2025). Scutellaria baicalensis ameliorates allergic airway inflammation through agonism and transcriptional regulation of TAS2Rs. J. Ethnopharmacol..

[56] Peng C., Zhang X., Zhou N., Hu T., Shen Y., Chen T.J., Liu Y., Cui H., Zhu S. (2024). Apigenin inhibits lipid metabolism of hepatocellular carcinoma cells by targeting the histone demethylase KDM1A. Phytomedicine.

[57] Alam F., Mohammadin K., Shafique Z., Amjad S.T., bin Asad M.H.H. (2022). Citrus flavonoids as potential therapeutic agents: A review. Phytother. Res..

[58] Qiu M., Liu J., Su Y., Guo R., Zhao B., Liu J. (2020). Diosmetin Induces Apoptosis by Downregulating AKT Phosphorylation via P53 Activation in Human Renal Carcinoma ACHN Cells. Protein Pept. Lett..

[59] Kim K., Leem J. (2022). Hispidulin Ameliorates Endotoxin-Induced Acute Kidney Injury in Mice. Molecules.

[60] Chaudhry G.E., Zeenia, Sharifi-Rad J., Calina D. (2024). Hispidulin: A promising anticancer agent and mechanistic breakthrough for targeted cancer therapy. Naunyn Schmiedebergs Arch. Pharmacol..

[61] Liu Z., Wang K., Jiang C., Chen Y., Liu F., Xie M., Yim W.Y., Yao D., Qian X., Chen S. (2024). Morusin Alleviates Aortic Valve Calcification by Inhibiting Valve Interstitial Cell Senescence Through Ccnd1/Trim25/Nrf2 Axis. Adv. Sci..

[62] Yao Y., Zuo X., Shao F., Yu K., Liang Q. (2024). Jaceosidin attenuates the progression of hepatic fibrosis by inhibiting the VGLL3/HMGB1/TLR4 signaling pathway. Phytomedicine.

[63] Liu J., Li S.M., Tang Y.J., Cao J.L., Hou W.S., Wang A.Q., Wang C., Jin C.H. (2024). Jaceosidin induces apoptosis and inhibits migration in AGS gastric cancer cells by regulating ROS-mediated signaling pathways. Redox Rep..

[64] Yan H., Wang P., Zhou Q., Dong X., Wang Q., Yuan Z., Zhai B., Zhou Y. (2024). Eupafolin hinders cross-talk between gastric cancer cells and cancer-associated fibroblasts by abrogating the IL18/IL18RAP signaling axis. Phytomedicine.

[65] Lee B.E., Park S.J., Kim G.H., Joo D.C., Lee M.W. (2024). Anti-inflammatory effects of eupatilin on Helicobacter pylori CagA-induced gastric inflammation. PLoS ONE.

[66] Wang L., Feng T., Su Z., Pi C., Wei Y., Zhao L. (2022). Latest research progress on anticancer effect of baicalin and its aglycone baicalein. Arch. Pharmacal Res..

[67] Chen H., Liu C., Zhan Y., Wang Y., Hu Q., Zeng Z. (2024). Alpinetin ameliorates bleomycin-induced pulmonary fibrosis by repressing fibroblast differentiation and proliferation. Biomed. Pharmacother..

[68] Rahmani A.H., Babiker A.Y., Anwar S. (2023). Hesperidin, a Bioflavonoid in Cancer Therapy: A Review for a Mechanism of Action through the Modulation of Cell Signaling Pathways. Molecules.

[69] Yue Y., Qian W., Li J., Wu S., Zhang M., Wu Z., Ma Q., Wang Z. (2020). 2′-Hydroxyflavanone inhibits the progression of pancreatic cancer cells and sensitizes the chemosensitivity of EGFR inhibitors via repressing STAT3 signaling. Cancer Lett..

[70] Tung M.C., Fung K.M., Hsu H.M., Tseng T.S. (2022). Correction: Discovery of 8-prenylnaringenin from hop (*Humulus lupulus* L.) as a potent monoacylglycerol lipase inhibitor for treatments of neuroinflammation and Alzheimer’s disease. RSC Adv..

[71] Xiong H.H., Lin S.Y., Chen L.L., Ouyang K.H., Wang W.J. (2023). The Interaction between Flavonoids and Intestinal Microbes: A Review. Foods.

[72] Wu K., Li Y., Ma K., Zhao W., Yao Z., Zheng Z., Sun F., Mu X., Liu Z., Zheng J. (2024). The microbiota and renal cell carcinoma. Cell. Oncol..

[73] De Morais E.F., de Oliveira L.Q.R., de Farias Morais H.G., de Souto Medeiros M.R., de Almeida Freitas R., Rodini C.O., Coletta R.D. (2024). The Anticancer Potential of Kaempferol: A Systematic Review Based on In Vitro Studies. Cancers.

[74] Ruan G.Y., Ye L.X., Lin J.S., Lin H.Y., Yu L.R., Wang C.Y., Mao X.D., Zhang S.H., Sun P.M. (2023). An integrated approach of network pharmacology, molecular docking, and experimental verification uncovers kaempferol as the effective modulator of HSD17B1 for treatment of endometrial cancer. J. Transl. Med..

[75] Zhu Q., Han Y., He Y., Fu Y., Yang H., Chen Y., Shi Y. (2023). Kaempferol Improves Breast Cancer-Related Depression through the COX-2/PGE2 Pathway. Front. Biosci..

[76] Pu Y., Han Y., Ouyang Y., Li H., Li L., Wu X., Yang L., Gao J., Zhang L., Zhou J. (2024). Kaempferol inhibits colorectal cancer metastasis through circ_0000345 mediated JMJD2C/β-catenin signalling pathway. Phytomedicine.

[77] Wang D., Chen J., Pu L., Yu L., Xiong F., Sun L., Yu Q., Cao X., Chen Y., Peng F. (2023). Galangin: A food-derived flavonoid with therapeutic potential against a wide spectrum of diseases. Phytother. Res..

[78] Zhou Y., Huang X., Yu H., Shi H., Chen M., Song J., Tang W., Teng F., Li C., Yi L. (2023). TMT-based quantitative proteomics revealed protective efficacy of Icariside II against airway inflammation and remodeling via inhibiting LAMP2, CTSD and CTSS expression in OVA-induced chronic asthma mice. Phytomedicine.

[79] Xu F., Wu Q., Li L., Gong J., Huo R., Cui W. (2021). Icariside II: Anticancer Potential and Molecular Targets in Solid Cancers. Front. Pharmacol..

[80] Huong N.T., Son N.T. (2023). Icaritin: A phytomolecule with enormous pharmacological values. Phytochemistry.

[81] Huang H., Wang J., Hussain S.A., Gangireddygari V.S.R., Fan Y. (2023). Gossypin exert lipopolysaccharide induced lung inflammation via alteration of Nrf2/HO-1 and NF-κB signaling pathway. Environ. Toxicol..

[82] Xiu Y., Su Y., Gao L., Yuan H., Xu S., Liu Y., Qiu Y., Liu Z., Li Y. (2023). Corylin accelerated wound healing through SIRT1 and PI3K/AKT signaling: A candidate remedy for chronic non-healing wounds. Front. Pharmacol..

[83] Xu S., Huang P., Yang J., Du H., Wan H., He Y. (2023). Calycosin alleviates cerebral ischemia/reperfusion injury by repressing autophagy via STAT3/FOXO3a signaling pathway. Phytomedicine.

[84] Song J., Ham J., Park S., Park S.J., Kim H.S., Song G., Lim W. (2023). Alpinumisoflavone Activates Disruption of Calcium Homeostasis, Mitochondria and Autophagosome to Suppress Development of Endometriosis. Antioxidants.

[85] Sharifi-Rad J., Quispe C., Imran M., Rauf A., Nadeem M., Gondal T.A., Ahmad B., Atif M., Mubarak M.S., Sytar O. (2021). Genistein: An Integrative Overview of Its Mode of Action, Pharmacological Properties, and Health Benefits. Oxidative Med. Cell. Longev..

[86] Li M., Yu Y., Xue K., Li J., Son G., Wang J., Qian W., Wang S., Zheng J., Yang C. (2023). Genistein mitigates senescence of bone marrow mesenchymal stem cells via ERRα-mediated mitochondrial biogenesis and mitophagy in ovariectomized rats. Redox Biol..

[87] Kaufman-Szymczyk A., Jalmuzna J., Lubecka-Gajewska K. (2025). Soy-derived isoflavones as chemo-preventive agents targeting multiple signalling pathways for cancer prevention and therapy. Br. J. Pharmacol..

[88] Chen Z., Ding W., Yang X., Lu T., Liu Y. (2024). Isoliquiritigenin, a potential therapeutic agent for treatment of inflammation-associated diseases. J. Ethnopharmacol..

[89] Hou Z., Wang Y., Chen S., Luo Z., Liu Y. (2024). Licochalcone A loaded multifunctional chitosan hyaluronic acid hydrogel with antibacterial and inflammatory regulating effects to promote wound healing. Int. J. Biol. Macromol..

[90] Yang L., Xiong J., Li S., Liu X., Deng W., Liu W., Fu B. (2023). Mitochondrial metabolic reprogramming-mediated immunogenic cell death reveals immune and prognostic features of clear cell renal cell carcinoma. Front. Oncol..

[91] Shen N., Wang T., Gan Q., Liu S., Wang L., Jin B. (2022). Plant flavonoids: Classification, distribution, biosynthesis, and antioxidant activity. Food Chem..

[92] Luo Y., Jian Y., Liu Y., Jiang S., Muhammad D., Wang W. (2022). Flavanols from Nature: A Phytochemistry and Biological Activity Review. Molecules.

[93] Teng H., Zheng Y., Cao H., Huang Q., Xiao J., Chen L. (2023). Enhancement of bioavailability and bioactivity of diet-derived flavonoids by application of nanotechnology: A review. Crit. Rev. Food Sci. Nutr..

[94] Li J., Tan G., Cai Y., Liu R., Xiong X., Gu B., He W., Liu B., Ren Q., Wu J. (2021). A novel Apigenin derivative suppresses renal cell carcinoma via directly inhibiting wild-type and mutant MET. Biochem. Pharmacol..

[95] Bories C., Lejour T., Adolphe F., Kermasson L., Couvé S., Tanguy L., Luszczewska G., Watzky M., Poillerat V., Garnier P. (2024). DCLRE1B/Apollo germline mutations associated with renal cell carcinoma impair telomere protection. Biochim. Biophys. Acta Mol. Basis Dis..

[96] Aiello P., Consalvi S., Poce G., Raguzzini A., Toti E., Palmery M., Biava M., Bernardi M., Kamal M.A., Perry G. (2021). Dietary flavonoids: Nano delivery and nanoparticles for cancer therapy. Semin. Cancer Biol..

[97] Fan Y., Hou T., Dan W., Liu T., Luan J., Liu B., Li L., Zeng J. (2020). Silibinin inhibits epithelial-mesenchymal transition of renal cell carcinoma through autophagy-dependent Wnt/β-catenin signaling. Int. J. Mol. Med..

[98] Takke A., Shende P. (2021). Magnetic-core-based silibinin nanopolymeric carriers for the treatment of renal cell cancer. Life Sci..

[99] Caparica R., Júlio A., Araújo M.E.M., Baby A.R., Fonte P., Costa J.G., Santos de Almeida T. (2020). Anticancer Activity of Rutin and Its Combination with Ionic Liquids on Renal Cells. Biomolecules.

[100] Mazurakova A., Koklesova L., Csizmár S.H., Samec M., Brockmueller A., Šudomová M., Biringer K., Kudela E., Pec M., Samuel S.M. (2024). Significance of flavonoids targeting PI3K/Akt/HIF-1α signaling pathway in therapy-resistant cancer cells—A potential contribution to the predictive, preventive, and personalized medicine. J. Adv. Res..

[101] Wang Y., Chen S., Sun S., Liu G., Chen L., Xia Y., Cui J., Wang W., Jiang X., Zhang L. (2020). Wogonin Induces Apoptosis and Reverses Sunitinib Resistance of Renal Cell Carcinoma Cells via Inhibiting CDK4-RB Pathway. Front. Pharmacol..

[102] Liu X., Zhang D., Hao Y., Liu Q., Wu Y., Liu X., Luo J., Zhou T., Sun B., Luo X. (2018). Cyanidin Curtails Renal Cell Carcinoma Tumorigenesis. Cell. Physiol. Biochem..

[103] Hsieh M.H., Tsai J.P., Yang S.F., Chiou H.L., Lin C.L., Hsieh Y.H., Chang H.R. (2019). Fisetin Suppresses the Proliferation and Metastasis of Renal Cell Carcinoma through Upregulation of MEK/ERK-Targeting CTSS and ADAM9. Cells.

[104] Jiang T., Liang Y., Ji Y., Xue Y. (2024). Fisetin enhances cisplatin sensitivity in renal cell carcinoma via the CDK6/PI3K/Akt/mTOR signaling pathway. Oncol. Lett..

[105] Bai Y., Xiong Y., Zhang Y.Y., Cheng L., Liu H., Xu K., Wu Y.Y., Field J., Wang X.D., Zhou L.M. (2022). Tangeretin Synergizes with 5-Fluorouracil to Induce Autophagy through MicroRNA-21 in Colorectal Cancer Cells. Am. J. Chin. Med..

[106] Lai Y., Wu W., Liang X., Zhong F., An L., Chang Z., Cai C., He Z., Wu W. (2023). Connexin43 is associated with the progression of clear cell renal carcinoma and is regulated by tangeretin to sygergize with tyrosine kinase inhibitors. Transl. Oncol..

[107] Singh B., Semwal B.C. (2024). A Compressive Review on Source, Toxicity and Biological Activity of Flavonoid. Curr. Top. Med. Chem..

[108] Ranawat P., Bakshi N. (2017). Naringenin; a bioflavonoid, impairs the reproductive potential of male mice. Toxicol. Mech. Methods.

[109] Zhang K., Dong R., Sun K., Wang X., Wang J., Yang C.S., Zhang J. (2017). Synergistic toxicity of epigallocatechin-3-gallate and diethyldithiocarbamate, a lethal encounter involving redox-active copper. Free Radic. Biol. Med..

[110] Bisol Â., de Campos P.S., Lamers M.L. (2020). Flavonoids as anticancer therapies: A systematic review of clinical trials. Phytother. Res..

[111] Li X., Xie E., Sun S., Shen J., Ding Y., Wang J., Peng X., Zheng R., Farag M.A., Xiao J. (2025). Flavonoids for gastrointestinal tract local and associated systemic effects: A review of clinical trials and future perspectives. J. Adv. Res..

[112] Van Veldhuizen P.J., Faulkner J.R., Lara P.N., Gumerlock P.H., Goodwin J.W., Dakhil S.R., Gross H.M., Flanigan R.C., Crawford E.D. (2005). A phase II study of flavopiridol in patients with advanced renal cell carcinoma: Results of Southwest Oncology Group Trial 0109. Cancer Chemother. Pharmacol..

